# Factors Controlling Soil Microbial Biomass and Bacterial Diversity and Community Composition in a Cold Desert Ecosystem: Role of Geographic Scale

**DOI:** 10.1371/journal.pone.0066103

**Published:** 2013-06-18

**Authors:** David J. Van Horn, M. Lee Van Horn, John E. Barrett, Michael N. Gooseff, Adam E. Altrichter, Kevin M. Geyer, Lydia H. Zeglin, Cristina D. Takacs-Vesbach

**Affiliations:** 1 Department of Biology, University of New Mexico, Albuquerque New Mexico, United States of America; 2 Department of Psychology, University of South Carolina, Columbia, South Carolina, United States of America; 3 Department of Biological Sciences, Virginia Technological Institute, Blacksburg Virginia, United States of America; 4 Department of Civil & Environmental Engineering, Pennsylvania State University, University Park, Pennsylvania, United States of America; 5 Department of Crop and Soil Science, Oregon State University, Corvallis Oregon, United States of America; Argonne National Laboratory, United States of America

## Abstract

Understanding controls over the distribution of soil bacteria is a fundamental step toward describing soil ecosystems, understanding their functional capabilities, and predicting their responses to environmental change. This study investigated the controls on the biomass, species richness, and community structure and composition of soil bacterial communities in the McMurdo Dry Valleys, Antarctica, at local and regional scales. The goals of the study were to describe the relationships between abiotic characteristics and soil bacteria in this unique, microbially dominated environment, and to test the scale dependence of these relationships in a low complexity ecosystem. Samples were collected from dry mineral soils associated with snow patches, which are a significant source of water in this desert environment, at six sites located in the major basins of the Taylor and Wright Valleys. Samples were analyzed for a suite of characteristics including soil moisture, pH, electrical conductivity, soil organic matter, major nutrients and ions, microbial biomass, 16 S rRNA gene richness, and bacterial community structure and composition. Snow patches created local biogeochemical gradients while inter-basin comparisons encompassed landscape scale gradients enabling comparisons of microbial controls at two distinct spatial scales. At the organic carbon rich, mesic, low elevation sites Acidobacteria and Actinobacteria were prevalent, while Firmicutes and Proteobacteria were dominant at the high elevation, low moisture and biomass sites. Microbial parameters were significantly related with soil water content and edaphic characteristics including soil pH, organic matter, and sulfate. However, the magnitude and even the direction of these relationships varied across basins and the application of mixed effects models revealed evidence of significant contextual effects at local and regional scales. The results highlight the importance of the geographic scale of sampling when determining the controls on soil microbial community characteristics.

## Introduction

Understanding the controls on the distribution of soil bacteria is essential for determining the functional capabilities of soil ecosystems and predicting their responses to environmental change, however, the complexity of these communities and their interactions with environmental characteristics have made generalizations difficult. Recently, high throughput sequencing technologies have facilitated the investigation of soil bacterial communities at local [1], regional [2], and global scales [3]. Species sorting related to environmental characteristics has been recognized as the most important mechanism controlling soil bacterial communities [4], [5] with pH identified as a master variable explaining significant portions of the variation in soil bacterial diversity and community structure at local [6], [7] and global [3], [8], [9] scales. However, while environmental factors have been identified as exerting primary control on soil bacterial distribution, on average approximately 50% of the variation in bacterial diversity and structure remains unexplained [5]. Additionally, very few examinations have been made of how controls on soil bacterial communities operate simultaneously at multiple scales to contrast local and regional drivers of bacterial diversity and community structure.

Investigating soil bacterial assemblages at local and regional scales in the McMurdo Dry Valleys, Antarctica, provides an opportunity to explore the controls on bacterial distribution in a low-complexity, extreme environment with simple food webs and an absence of plant-soil interactions. The Dry Valleys comprise the largest ice free zone in continental Antarctica [10] and represent one of the coldest and driest terrestrial environments on earth. The communities of eukaryotes found in these soils reflect the harsh environmental conditions with an absence of higher plants and limited protozoan [11], [12] and invertebrate [13], [14], [15] diversity.

In contrast to the limited eukaryotic diversity, recent lines of evidence support the existence of an indigenous and diverse microbial community in Dry Valley soils. Sequencing of the bacterial 16 S rRNA gene has revealed significant diversity in these soils at individual locations and basins [16], [17], [18], [19] and across the landscape [20], [21], [22]. A recent review of sequence data has shown the presence of 13 bacterial phyla in Dry Valley soils [23]. A few studies have performed more extensive sampling identifying altitude, pH, soil moisture, soil organic matter (SOM), total nitrogen, C:N ratio, and electrical conductivity as correlated with bacterial community structure and diversity [24], [25], [26]. Thus, these soil bacterial communities have received some attention to date, however, a spatially intensive, deep sequencing effort stratified at multiple spatial scales and coupled with edaphic characterization would advance our understanding of geographic and environmental drivers of microbial biodiversity.

The goal of this study was to determine the controls on the microbial biomass and bacterial richness and structure of McMurdo Dry Valley soil communities at local and regional scales. At each of six sites located in the major basins of Taylor and Wright Valley dry mineral soil samples and samples associated with snow patches were collected to capture local and inter-basin regional scale abiotic and biotic variation. Snow patches are a significant source of water in this desert environment, have been shown to alter metazoan communities and local biogeochemistry [27], and may serve as resource islands of enhanced nutrient cycling and availability. The specific objectives of this study were to: 1) determine if abiotic gradients control three key bacterial characteristics; microbial biomass, diversity, and community structure and composition in the Dry Valleys, and 2) to understand how these controls vary at local and regional scales to test the scale dependence of these relationships in a low-complexity ecosystem.

## Materials and Methods

### Site Description

The McMurdo Dry Valleys, Victoria Land, Antarctica (77° 30' S, 163° 00' E) comprise one of the coldest and driest environments on earth. Air and surface soil temperatures in this ice-free polar desert average ∼−20°C, with extremes ranging from −60 to 25°C on the soil surface [28]. Precipitation in the form of snow ranges from 3 to 50 mm water equivalent annually [29] with sublimation rates exceeding precipitation inputs [30]. Mineral soils are among the harshest Dry Valley habitat types with limited moisture, low SOM, variable conductivity, and high pH and UV radiation inputs. These soils are primarily classified as Anhyorthels and Anhyturbels, are underlain by permafrost within a meter of the surface, and originated as glacial tills with ages varying from 10–1000 KY [31]. Soils range in salinity (20 to >7000 µS cm^−1^) [31], [32], [33] and are low in SOM (mean 0.03% dry mass) [32], [34]. The sources of moisture to these soils are transient snow fall events, snow patches from winter precipitation and redistribution of snow from the polar plateau [27], and salt deliquescence in saline environments [35].

**Figure 1:**
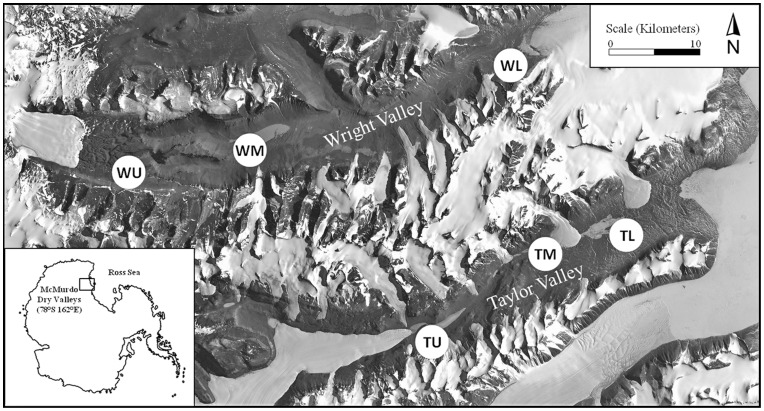
Site map showing the six sampling sites located in the Taylor and Wright Valleys.

A total of six sites were sampled, three each in Taylor Valley and Wright Valley. Prior to commencing our study, an Environmental Impact Statement was prepared by the United States Antarctic Program Office of Polar Environment, Health and Safety. No special permissions or permits were required to access our sampling locations and to perform our field activities because our research did not occur within any Antarctic Specially Protected or Managed Areas in accordance with the Protocol on Environmental Protection to the Antarctic Treaty. No endangered or protected species were involved in this study. Sites were located in the major hydrological basins of the lower, middle, and upper portions of Taylor and Wright Valley. Sites included the Lake Fryxell (Taylor Lower, TL), Lake Hoare (Taylor Middle, TM), Lake Bonney (Taylor Upper, TU), Lake Brownworth (WL), Lake Vanda (WM), and Labyrinth (WU) basins (Figure 1). Each site was associated with one seasonal snow patch located on the north-facing slope of the valley. Twelve samples were collected from each site in December 2009 along three transects from fixed points starting in exposed soils and extending to the edge of the snow patch representing a gradient from dry exposed soil to wet soil at the edge of the snow patch. In addition, four samples per site were collected from exposed soils at locations upslope from snow patches for a total of 16 samples per site and a total of 96 samples from all six sites. For each sample, the soil was aseptically collected to a depth of 10 cm from the top of the soil or until ice cement was encountered (the majority of soils were thawed to 10 cm depth during this period). Samples (∼250 g) were taken with sterilized scoops, sealed in sterile bags, and returned to the laboratory where they were sub-sampled under a laminar flow hood and sorted to isolate the fine soil fraction (<2 mm) from pebbles and cobble before further analysis.

### Basic Edaphic Characteristics

Soil moisture was determined gravimetrically on 25 g of fresh soil material after drying for 24 hr at 105°C and is reported as water content (WC, mg-water g-soil^−1^). Soil pH was estimated on 1∶2 soil/deionized water extracts using an Orion pH probe. Conductivity of 1∶5 soil/water extracts was measured with a Yellow Springs Instrument 3100 conductivity meter. Total nitrogen (TotN, µg-N g-soil^−1^) and soil organic carbon were estimated from ∼300 mg of ground, dried, and acidified samples using a FlashEA 1112 NC Elemental Analyzer (CE Elantech, Lakewood, NJ, USA). Soil organic carbon was converted to soil organic matter assuming carbon to be 45% of SOM (SOM, µg-C g-soil^−1^). The soluble ions chloride (Cl, µg-Cl g-soil^−1^) and sulfate (SO_4_, µg- SO_4_ g-soil^−1^) were measured using 1∶5 soil/deionized water extracts and analysis by standard ion chromatography methods (Dionex). Nitrate-N (NO_3_−N, µg-N g-soil^−1^) and ammonium-N (NH_4_−N, µg-N g-soil^−1^) were measured using a 1∶5 soil/2 M KCl extract and analyzed using standard colorimetric methods on a Lachat QuikChem 8500 Flow Injection Analyzer (Lachat Instruments, Loveland, CO, USA). Microbial biomass carbon (MBC, µg-MBC g-soil^−1^) was determined as reported previously [36].

### DNA Extraction, Sequencing, and Sequence Analysis

Soils were sub-sampled for molecular analysis and stored in sucrose lysis buffer [37] at −20°C until extraction of 0.7 g of soil with the cetyltrimethylammonium bromide (CTAB) method as described by Mitchell & Takacs-Vesbach [38]. Barcoded amplicon pyrosequencing of 16 S rRNA genes was performed as described previously [39], [40] using universal bacterial primers 939F 5′ TTG ACG GGG GCC CGC ACA AG and 1492R 5′-GTT TAC CTT GTT ACG ACT T-3′. Briefly, 100 ng of DNA per sample was amplified in triplicate by a single step PCR to create 16 S rRNA gene amplicons containing the Roche-specific sequencing adapters and a barcode unique to each sample. Amplicons were purified using Agencourt Ampure beads and combined in equimolar concentrations. Pyrosequencing was performed on a Roche 454 FLX instrument using Roche titanium reagents and titanium procedures.

The 16 S rRNA gene sequences were quality filtered, denoised, screened for PCR errors, and chimera checked using default parameters in AmpliconNoise and Perseus [41]. The Quantitative Insights into Microbial Ecology (QIIME) pipeline was used to analyze the 16 S rRNA gene sequence data [42]. Unique 16 S rRNA gene sequences or operational taxonomic units (OTUs) were identified by the 97% DNA identity criterion using UCLUST [43]. A representative sequence was picked from each OTU and aligned using the PyNAST aligner [44] and the Greengenes core set [45] and given taxonomic assignments using the Ribosomal Database Classifier program [46]. All measures of community structure (observed OTUs and Chao1 richness per sample) were performed with randomly selected subsets of 750 sequences per sample to standardize for varying sequencing efforts across samples. Raw sequence data from this study are available through the NCBI Sequence Read Archive as SRP018437. The individual sff files from this study were assigned the accession numbers SAMN01909167 through SAMN01909254 under Bioproject PRJNA188346.

### Data Analysis to Identify Controls on Bacterial Distribution

To reduce multicollinearity and focus the analyses on the variables most likely to be important in controlling bacterial communities we selected five of the nine edaphic variables with variance inflation factors under 4.00 for all but one variable (MBC/SOM basin mean  = 6.49) which was lower than the recommended upper limit of ten [47]. The five predictors of MBC and 16 S rRNA gene richness selected were WC, pH, SOM, SO_4_, and NH_4_−N. To linearize relationships and to aid in the interpretation of model estimates, the predictor variables were scaled as follows: the basin mean sample distance from the snow patch was subtracted from the distance for each sample and divided by the basin standard deviation. All other variables were normalized by taking the natural log (except pH) and centering by subtracting the mean across all samples. This centering allows the intercept, which is an important component of mixed models, to be interpreted as the expected value of the outcome for the average sample. The effects of proximity to snow patch on soil moisture, soil moisture on edaphic characteristics, and edaphic characteristics on microbial biomass and diversity were tested at local and regional scales using mixed effects models implemented in R 2.14 [48] with the nlme package [49]. Graphical examination of the bivariate relationship between the predictor and outcome typically revealed more complex patterns when examined across basins and at the regional scale. These patterns suggest the use of a more complex specification of the mixed effects model to evaluate the contextual effects. Contextual effects are defined as relationships between predictor and outcome which are dependent upon geographical context [50], relationships which are evident in these figures.

**Figure 2:**
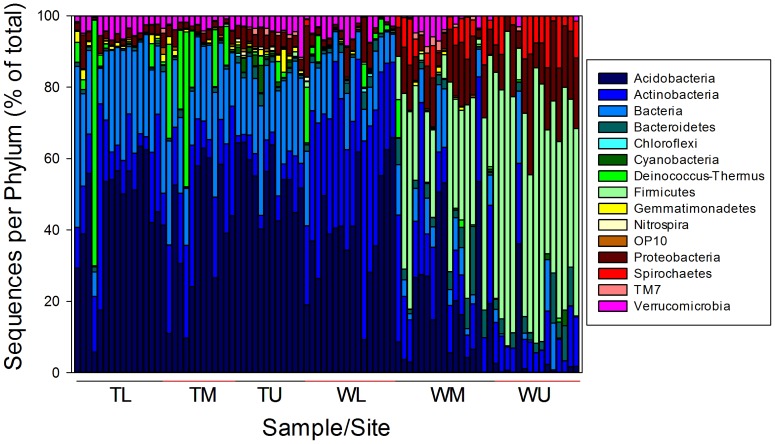
The phyla level taxonomy for each sample expressed as the percentage of the total sequences.

**Figure 3:**
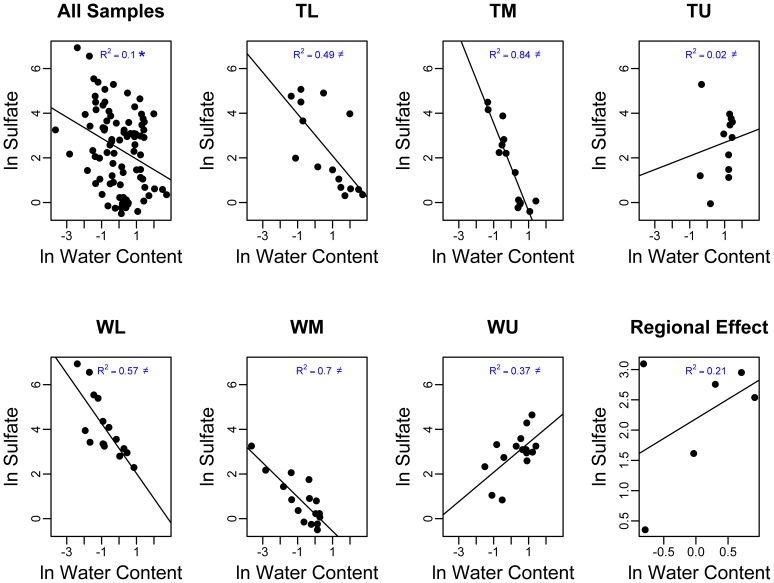
The ln of the water content (mg water g^−1^ dry soil) versus the ln of the sulfate values (ug sulfate g^−1^ dry soil) for all of the samples (pane 1), at the six sites (panes 2–7), and regional effects (pane 8). Significant relationships have been noted for panes 1 and 8 (*) and a ≠ in panes 2–7 indicates that the within site relationships are statistically different across sites (i.e. a local contextual effect was found).

**Figure 4:**
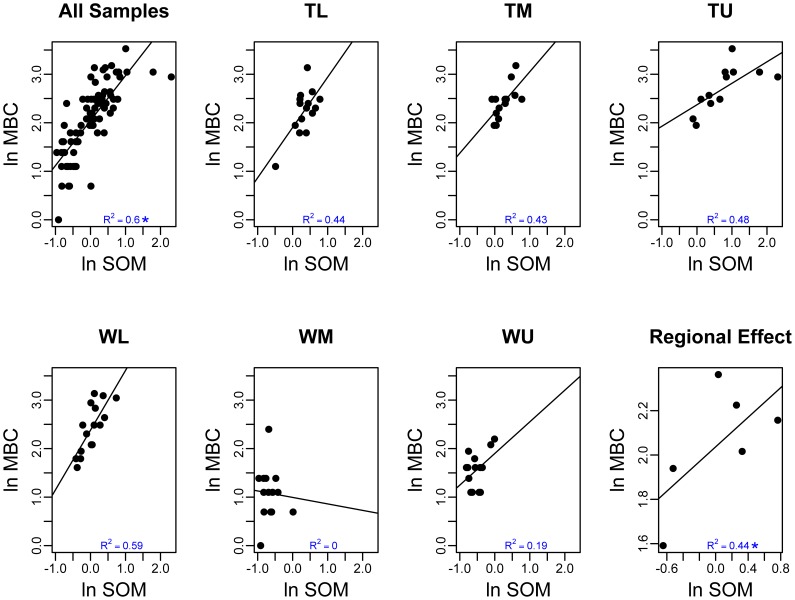
The ln of the soil organic matter (ug SOM g^−1^ dry soil) versus the ln of the microbial biomass carbon (ug MBC g^−1^ dry soil) for all of the samples (pane 1), at the six sites (panes 2–7), and regional effects (pane 8). Significant relationships have been noted for panes 1 and 8 (*) and a ≠ in panes 2–7 indicates that the within site relationships are statistically different across sites (i.e. a local contextual effect was found).

The predictor-response relationships across these variables were decomposed into three components: 1) the average local, 2) local contextual effects, and 3) regional effects within each predictor/response variable pair. (1) At the local level we tested the average relationship between the predictor and response, which, when significant, infers that on average this relationship is non-zero (average local effects). (2) Local contextual effects were investigated by testing for variability in the slope of the predictor/response relationships between basins, suggesting that the predictor-response relationship differs between study sites (local contextual effects). (3) At the regional level we tested for relationships between the basin means of the predictor and the basin means of the response variable adjusted for differences across basins due to the within basin effect (regional effects). This removes the average local level relationship between the predictor and response variables and tests for underlying differences that affect the predictor/response variable relationships at a larger scale [50]. For example, if a positive average local relationship existed between soil organic matter and microbial biomass, the regional effect would ask if there is an additional contextual effect of SOM on MBC when the relationship is examined across regions (as depicted in the last pane of Figures 2–4). The regional effect could, for example, be due to the influence of factors such as varying quality of SOM across a region. A significant regional effect suggests that the processes connecting the predictor and response are contextual, operating differently at various scales. Due to the limited number of basins available for analysis we used an alpha level of 0.10 to test local contextual effects which provides a tradeoff between power and type I error [51]. (See Text S1 for a detailed description of contextual effects and the mixed effects modeling.).

Standardized OTU tables from the 16 S rRNA gene sequence data were imported into the Vegan package in R [52] for further analysis. Bray-Curtis dissimilarity matrices were created from OTU tables and were ordinated using nonmetric multidimensional scaling (NMDS). The envfit function was used to perform vector fitting of the environmental variables onto the NMDS ordinations using 10,000 permutations to assess the significance of the fit. The adonis function, which performs analysis of variance using distance matrices, was used to test the overall and pair-wise dissimilarity of the community structure of samples from different sites [52]. An indicator species analysis (ISA, *indicspecies*) performed on the standardized data was used to identify OTUs indicative of specific sites [53], [54]. The five most significantly correlated indicator species for each site were added to the NMDS ordination discussed above using the orditorp function in Vegan [52].

## Results

### Sequencing, 16 S rRNA Gene Richness, and Taxonomy of Dry Valley Soil Bacterial Communities

Pyrosequencing of the 16 S rRNA gene resulted in 435,722 sequences from 88 samples following the removal of low quality sequences and chimeras, and denoising (eight samples had an insufficient number of DNA sequences). The number of sequences per sample ranged from 1,071 to 17,544 with an average sequence count of 4,951. These sequences represented 4,128 OTUs (97% similarity) and rarefaction curves show that a majority of the samples approached asymptote (Figure S1).

The bacterial community composition was relatively consistent within sites but varied dramatically across sites (Figure 2). At the phyla level the majority of sequences across sites were found in the Acidobacteria (33%) followed by Actinobacteria (17%), Firmicutes (15%), unidentified bacteria (15%), Proteobacteria (7%), Deinococcus-Thermus (3%), Verrucomicrobia (3%), Bacteroidetes (2%), and Spirochaetes (2%). The three Taylor Valley sites were consistently dominated by Acidobacteria (46%), unassigned bacteria (23%), Actinobacteria (14%), and Deinococcus-Thermus (6%). The WL site had a similar composition as the TL sites with the exception of a higher proportion of Acidobacteria (35%) and fewer Actinobacteria (40%). Sequences from the WM and WU sites contained fewer Acidobacteria (12%) and Actinobacteria (13%) and more Firmicutes (43%), Proteobacteria (13%), and Spirochaetes (6%) (Figure 2).

### Local and Regional Edaphic Gradients

Results of the mixed effects model found a significant (*P*<0.05) negative relationship for the average local effect of distance from the snow patch edge on soil moisture confirming that the sampling design successfully captured local moisture gradients. However, there was also a significant local contextual effect on soil moisture (*P*<0.10): the gradient of high soil moisture near the snowpack to low soil moisture distant from the snowpack was strong in three basins (TL, TM, WL), weak in two basins (TU, WU) and weakly reversed in the basin with lowest soil water content (WM). Significantly less soil moisture (*P*<0.05) was also found in exposed soils than in snow patch associated soil samples (Figure S2).

The effects of soil moisture on edaphic chemical characteristics varied between parameters and sites. There was no evidence of statistically significant relationships for the average local effect of soil moisture on any of the five edaphic characteristics analyzed. However, there was evidence of significant (*P*<0.10) local contextual effects for the relationships between soil moisture and pH and SO_4_ indicating large differences in these relationships among basins. The effect of moisture on SO_4_ ranged from strongly positive to moderately strong and negative (Figure 3) while the effect of moisture on pH was moderately strong and positive in two basins, negative in two basins, and negligible in two other basins. A significant (*P*<0.05) regional effect was also found for SOM such that the basins with higher average moisture content had higher levels of SOM.

### Local and Regional Edaphic Gradient Relationships to Bacterial Community Characteristics

The relationships between local and regional edaphic characteristics and gradients and bacterial community properties were complex and varied. Microbial biomass was significantly related with edaphic characteristics at both local and regional scales (Table 1). Average local effects were found for the relationship between microbial biomass and three edaphic characteristics, with a negative relationship to SO_4_ (*P*<0.05), and positive relationships to soil moisture (*P*<0.05) and SOM (*P*<0.05, Figure 4). No evidence was found of significant local contextual effects. At the regional scale significant positive relationships were observed for MBC and pH (*P*<0.05), NH_4_−N (*P*<0.05), and SOM (*P*<0.05, Figure 4), demonstrating that the effect of these edaphic characteristics was evident at the regional scale.

**Table 1:** Relationships between ln normalized edaphic variables and ln normalized MBC.

Measures	Soil Moisture	SOM	pH	NH_4_	SO_4_
**Average Local Effect**	0.198 (0.044)*	0.622 (0.120)*	−0.252 (0.134)	0.036 (0.044)	−0.112 (0.032)*
**Local Contextual Effect (SD)**	MNS	MNS	MNS	MNS	MNS
**Regional Effect**	0.180 (0.134)	0.460 (0.214)*	1.10 (0.304)*	0.650 (0.159)*	0.400 (0.201)
**Intercept**	2.06 (0.209)	2.04 (0.087)	2.06 (0.144)	2.06 (0.111)	2.07 (0.201)
**SD of Rand. Intercept**	0.490*	0.181*	0.326*	0.238*	0.472*
**SD of Residual**	0.40	0.389	0.442	0.453	0.42

Significance at P<0.05 (or P<0.10 for local contextual effects) is indicated by *, MNS indicates the model was not significant.

**Figure 5:**
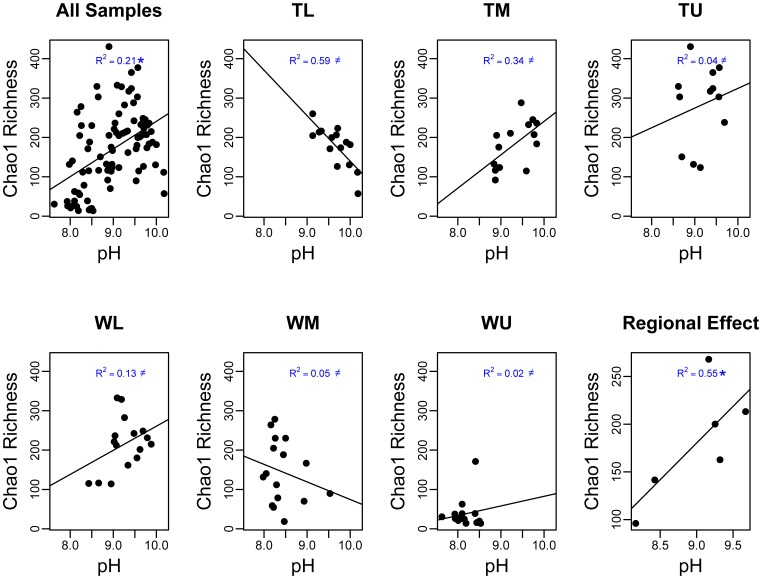
The soil pH versus the Chao1 estimates of 16 S rRNA gene richness (per 750 16 S rRNA gene sequences) for all of the samples (pane 1), at the six sites (panes 2–7), and regional effects (pane 8). Significant relationships have been noted for panes 1 and 8 (*) and a ≠ in panes 2–7 indicates that the within site relationships are statistically different across sites (i.e. a local contextual effect was found).

Relationships between edaphic characteristics and 16 S rRNA gene bacterial richness estimates were more complex than those of MBC (Table 2). A single average local effect was significant (NH_4_), while local contextual effects were significant (*P*<0.05) for the regression of bacterial richness on four of the five edaphic variable relationships: pH (Figure 5), SO_4_, soil moisture, and NH_4_. At the regional scale bacterial richness was positive and significantly correlated with pH (Figure 5) and SOM.

**Table 2:** Relationships between edaphic variables and Chao1 richness estimates.

Measures	Soil Moisture	SOM	pH	NH_4_	SO_4_
**Average Local Effect**	15.2 (12.3)	−44.5 (20.4)	13.0 (30.8)	15.0 (6.34)*	−11.6 (8.63)
**Local Contextual Effect (SD)**	29.6*	MNS	64.2*	24.2*	19.5*
**Regional Effect**	−10.7 (44.3)	168 (42.0)*	101 (42.0)*	29.5 (38.2)	31.8 (33.2)
**Intercept**	174 (29.5)	169 (17.9)	176 (19.4)	173 (27.4)	179 (33.2)
**SD of Rand. Intercept**	84.0*	49.6*	48.4*	74.7*	96.3*
**SD of Residual**	62.5	66.7	65.2	61.9	61.3

Significance at P<0.05 (or P<0.10 for local contextual effects) is indicated by *, MNS indicates the model was not significant.

**Figure 6:**
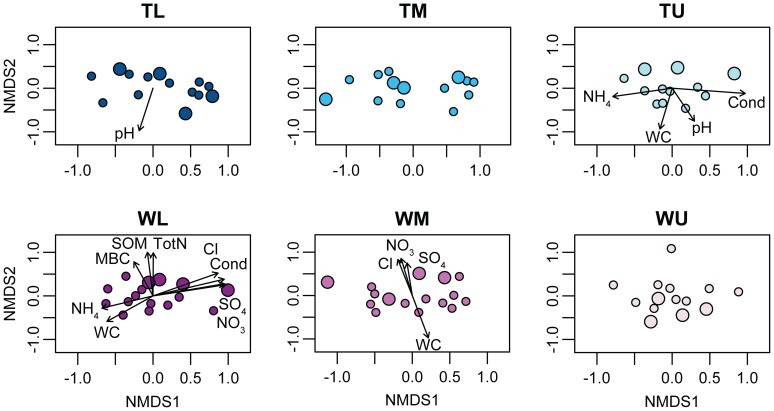
Nonmetric multidimensional scaling ordinations of the Bray-Curtis distance matrices created from the OTU (97% DNA identity) tables for each of the six sites with vector overlays of the significantly correlated normalized edaphic characteristics. (Cl = chloride, Cond = electrical conductivity, MBC = microbial biomass carbon, TotN = total nitrogen, WC = water content, pH = soil pH, SO_4_ =  sulfate, SOM = soil organic matter). The large circles represent control samples collected outside of the influence of the snow patches and the small circles represent snow patch associated samples.

**Figure 7:**
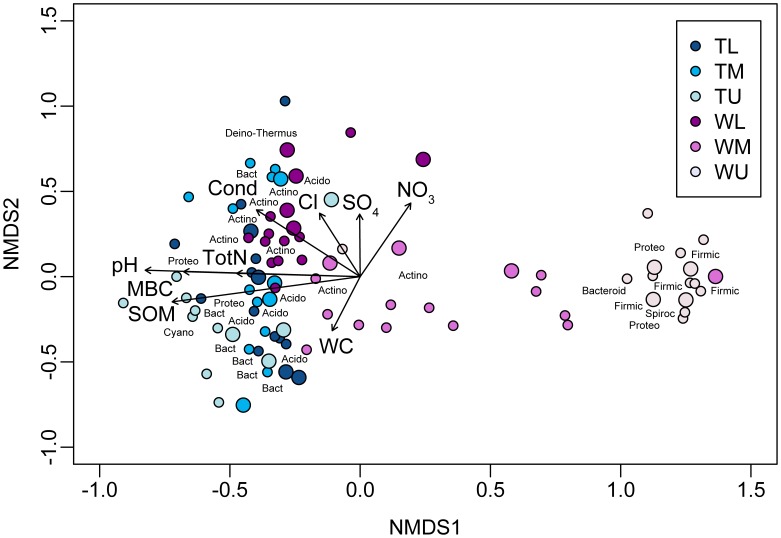
Nonmetric multidimensional scaling plots of the Bray-Curtis distance matrices created from the OTU (97% DNA identity) table for all six sites combined with the five most significantly correlated indicator species for each site and a vector overlay of the significantly correlated normalized edaphic characteristics. (Cl = chloride, Cond = electrical conductivity, MBC = microbial biomass carbon, TotN = total nitrogen, WC = water content, pH = soil pH, SO_4_ =  sulfate, SOM = soil organic matter). The large circles represent control samples collected outside of the influence of the snow patches and the small circles represent snow patch associated samples.

The relationships between bacterial community structure and edaphic characteristics were explored both within and across sites using NMDS ordination of community dissimilarities and a vector overlay of the significant edaphic parameters. As with the relationships between edaphic characteristics and microbial biomass and bacterial richness, bacterial community structure was significantly (*P*>0.05) related with different edaphic characteristics at different sites and spatial scales. Relationships were absent at two sites (TM, WU), limited at three sites (TL, TU, and WM), and complex at a single site (WL, Figure 6). Water content was the only variable significantly correlated with bacterial community structure at more than two sites. Additionally, the bacterial community structure of exposed soil samples did not differ significantly (*P*>0.05) from that of the snow patch associated samples (Figure 6). At the regional scale, eight of the nine edaphic variables and MBC were significantly (*P*<0.05) related with the overall bacterial community structure. Salt related variables (conductivity, Cl, SO_4_, and NO_3_) grouped together and were negatively related to soil moisture (Figure 7). A third distinct grouping consisted of resource related variables (TotN, SOM, and MBC) and pH. Results from the adonis test indicate a significant (*P*>0.05) global difference between the site groupings and significant pairwise (*P*>0.05) differences for all comparisons with the exception of the TL/TM sites.

A large number of indicator species were identified for each site ranging from 16 (WU) to 88 (TU). The five most significantly correlated indicator species for each site reflected the site level taxonomic distributions discussed above. Indicator species from the Taylor Valley sites were dominated by unassigned bacteria (6), Acidobacteria (4), Gammaproteobacteria (2), and one each of Actinobacteria, Cyanobacteria, and Deinococcus-Thermus (Figure 7). The WL site indicator species were dominated by Actinobacteria (4) and one Acidobacteria while the WM and WU sites contained Firmicutes (4), Actinobacteria (2), Gammaproteobacteria (2), and one each of Bacteroidetes and Spirochaetes (Figure 7).

## Discussion

This study utilized edaphic characterization, microbial biomass estimates, and extensive sequencing of a large number of samples to identify the environmental controls on soil microbial communities in the Dry Valleys and to more generally test the scale dependence of these relationships. The results clearly point to complex interactions with underlying mechanisms even in a low-complexity environment.

### Implications for Understanding Controls on Dry Valley Soil Bacterial Communities

The snow patches studied increased soil water content in patch related versus control samples and created moisture gradients in spite of the extremely high local sublimation rates [30]. This is consistent with a previous study of subnivian soils in the Dry Valleys which found increased soil moisture under seasonal snow patches [27]. However, the moisture gradients did not produce predictable edaphic gradients: significant average local effects were absent and significant local contextual effects were found for the relationship between soil moisture and pH and SO_4_. The absence of snow patch related chemical gradients was unexpected: in non-Antarctic environments snow patches are known to produce gradients of major ions, nutrients, and pH due to water mediated chemical and microbial weathering of parent material [55]. The lack of a SOM gradient was particularly interesting as surface soils in the Dry Valleys that receive persistent water inputs typically support cyanobacterial production elevating SOM [32], [56], [57]. Examination of taxonomic data revealed that cyanobacteria were absent from the majority of the samples and were not observed to consistently decrease in abundance with distance from the snow patches. This suggests that while snow patch related gradients did occur, the mechanisms and transport processes vary within individual basins and may be to ephemeral to predictably support primary production. These findings underscore the between basin variability which may result from the differences in parent geology [58], [59], glacial till sequences [60], [61], soil texture, and historic legacies [62] underlying snow patches from different basins of the McMurdo Dry Valleys.

The within site consistency and between site variation in phyla level bacterial community composition found in this study suggests that regional factors broadly dictate the base taxonomic composition of a given site. The observed taxonomy of the Taylor Valley and WL sites are consistent with soil communities found in other ecosystems with a prevalence of Acidobacteria, Actinobacteria, and unassigned bacteria, however, fewer Proteobacteria and Bacteroidetes and more Verrucomicrobia were found than in typical soils [63]. At the WM and WU sites the composition was much different than that observed in other soils with a dominance of Firmicutes, a relatively large proportion of Spirochaetes, and an increase in Proteobacteria and Bacteroidetes [63].

Previous investigations of Dry Valley soil bacterial taxonomy have shown considerable variability. The dominant phyla present at individual sites have included Actinobacteria [22], [24], [64], Acidobacteria [17], and Bacteroidetes [26] with Proteobacteria, Deinococcus-Thermus, Gemmatimonadetes, Firmicuties and Verrucomicrobia also commonly found. While methodological inconsistencies may explain some of these differences, in general studies that used the same methods at different sites have found evidence of significant phyla level variation among sites [17], [22], [26] suggesting the variation is environmentally driven. A recent study that utilized pyrosequencing (but with a limited number of samples and sequence reads per sample) found relatively similar communities at the phyla level but distinct communities at finer taxonomic resolution at four geographically disparate sites [24]. One of these sites was located in close proximity to our WU site but had a very different community composition. It is possible that the use of different primer sets or the disparity in sequencing depth (466 reads versus 70,600 reads in our study) may be responsible for the observed differences in community composition.

The relationships between environmental characteristics and microbial biomass, 16 S rRNA gene richness, and bacterial community structure and composition were highly complex at both spatial scales. Some of the relationships between MBC and edaphic characteristics were predicted. At the local scale the significant positive relationships between MBC and SOM and soil moisture have been observed previously along Dry Valley streams [36], and indicate that snow patches are operating as resource islands providing suitable habitat for microbial growth. A strong relationship between SOM and microbial biomass has been observed in many ecosystems and has been summarized in review papers [63], [65]. Dry Valley SOM values, however, are approximately two orders of magnitude lower than in other soils, and average MBC to SOM ratios are significantly higher (*P*<0.05, data not shown) than in other ecosystems [66] likely resulting from the absence of higher plants which contribute litter inputs containing refractory structural materials in other ecosystems. The negative and significant average local relationship between MBC and SO_4_ suggests increases in salt related variables negatively influence soil microbes, a pattern observed for invertebrates in this ecosystem [33]. Additionally, the absence of significant local contextual effects suggests that at the within basin level the controls on MBC are similar. However, the significant regional effects for three of the variables (SOM, pH, and NH_4_) suggest that the processes connecting these edaphic variables and MBC operate differently at different scales.

The controls on bacterial richness in the Dry Valleys demonstrate even greater variability and site specificity than the controls on MBC. This is evidenced at the local level by a single significant average local effect and significant local contextual effects for four of the five bacterial richness versus edaphic variable relationships. Evidence of significant regional effects for SOM and pH on bacterial richness provide further evidence that the processes connecting these variables operate differently at local and regional scales. At the regional level resource supply appeared to positively influence bacterial richness, with a positive relationship for SOM. Similar positive relationships have been observed in Antarctic soils [25] while unimodal relationships have been observed between microbial diversity and resource supply in other soils [67]. These unimodal relationships are unlikely in the Dry Valleys as SOM levels are too low to drive competitive exclusion processes which create the declines in diversity at the upper end of unimodal diversity/productivity relationships [68]. We found that soil pH was also positively related to bacterial richness at the regional scale, which was of interest because although this master variable controls bacterial diversity at many scales [3], [7], in other studies, diversity typically begins to decline above pH 7, but ranges in our sites from pH 8 to 10.

The relationships between bacterial community structure and edaphic characteristics also varied dramatically across sites and spatial scales. At the local scale, the limited consistency in the correlations between edaphic characteristics and community structure (only two edaphic variables were correlated with community structure at more than one site) again strongly suggests that the controls on community structure are highly contextual. At the regional scale the wide variety of significant correlations between edaphic characteristics and community structure suggests the large regional differences in edaphic characteristics are likely responsible for generating community differences. Of the four most well correlated edaphic variables, pH, SOM, MBC, and conductivity, pH is known to be a master variable controlling community structure in this [25] and other ecosystems through a wide variety of potential mechanisms [3] and conductivity has been shown to be important in structuring Dry Valley communities [24], [26].

The Taylor Valley sites had a higher proportion of Acidobacteria than soils from other environments [63]. Although these sites are the most soil organic matter rich in the Dry Valleys they contain orders of magnitude less organic matter than is typically found in other soils confirming that Acidobacteria thrive in oligotrophic environments [69]. Furthermore, the Acidobacteria observed in these soils were also almost exclusively from the G4 and G6 groups which are known to be more abundant at high soil pH [70], correlating well with the elevated pH found in the Taylor Valley sites. The abundance of Actinobacteria in the Taylor Valley sites is also consistent with the prevalence of these organisms in high pH environments [3]. The presence of Cyanobacteria at the TU site both as an indicator species and as evidenced in the bulk taxonomic data also correlate well with the high soil moisture at this location, suggesting conditions at this site promote *in-situ* primary production. The prevalence of Firmicutes at the WU site is likely due to the ability of these organisms to live in harsh environments as this site had low SOM, percent water content, and nutrients and is located at 3000 meters ASL, 2000 meters higher than the next highest site (TU). This high altitude subjects these organisms to increased UV input and lower temperatures, likely selecting for organisms with gram-positive cell walls and spore forming ability which lend resistance to desiccation stress and harsh environmental conditions [71], [72]. While the taxonomic composition at the various sites fits well with the observed edaphic characteristics, it does not necessarily explain the contextual effects discussed above. For example, the structure (Figure 7) and taxonomic composition (Figure 2) of the TL and TM sites was very similar (these sites were not significantly different from one other in the Adonis analysis). However, the relationship between richness and pH at these sites was different with an increase in richness with pH at the TM site and a decrease at the TL site.

The complex relationships between edaphic characteristics and microbial biomass, 16 S rRNA gene richness, and bacterial community structure and composition indicate that controls on these microbial communities are highly variable and scale dependent, even in a region typified by a low-complexity ecosystem with no higher plants, very low invertebrate diversity, and relatively uniform climatic conditions. The scale related importance of environmental factors in structuring communities has been noted in the Dry Valleys for nematodes [32] and in salt marsh ecosystems for bacteria [73]. In both studies the scale dependence was suggested to be a function of greater total environmental variability at larger scales. While this appears to be true for some variables in this study (SOM, pH), other variables exhibit a wide range of values at individual sites (WC, salt related variables). Thus, in this ecosystem it appears that some edaphic factors exert control on microbial communities regardless of scale while underlying characteristics such as parent geology or glacial till sequence moderate other factors. Other potentially complicating factors should be noted: it is possible that some of the 16 S rRNA gene sequences represent organisms that are no longer viable, and it is likely that other unmeasured edaphic, environmental, and historic factors interact to create these contextual effects.

### Broad Implications

The McMurdo Dry Valleys are a microbially dominated, low complexity ecosystem; attributes which make it an ideal natural laboratory to test the controls on microbial life with generalizable implications for other ecosystems. A significant body of research over the past several decades has conclusively demonstrated that microbial communities are not randomly distributed but show distinct spatial patterns of biomass [63], diversity [9], [74], and community structure [75], [76], patterns which are in agreement with observations from this study of Dry Valley soil bacteria. Considerable effort has been directed toward understanding the mechanisms underlying these patterns, much of which has been borrowed from the literature developed for metacommunities which identifies four potential processes driving diversity and community structure patterns: patch dynamics, neutral processes, species sorting, and mass-effects [77]. Of these potential processes, species sorting driven by localized abiotic and biotic interactions, which select for a subset of the metacommunity, has been shown to be the most important factor controlling microbial communities in a wide variety of individual studies [78], [79]. Recent reviews have also found strong evidence of species sorting by environmental selection in approximately 93% of the studies reviewed [5]. Evidence of the importance of mass effects related to dispersal processes and limitation are also apparent [5], [80], however, these effects are found less frequently and are generally weaker than those of species sorting [5]. These general conclusions from reviews of existing studies are in agreement with our finding of significant relationships between environmental factors and soil bacterial communities, widely varying taxonomic structure at spatially and edaphically distant sites, and strongly suggest that these communities are active and responding to environmental conditions.

While the importance of environmental selection in structuring microbial communities is clear, the vast majority of such studies have investigated these effects at a single scale, ranging from the local to global, but few if any studies have investigated the importance of microbial species sorting simultaneously at multiple spatial scales. This is potentially problematic as the importance of scale to understanding fundamental patterns in ecology has been noted previously [81] with species richness patterns identified as being particular susceptible to issues of scale [82]. The extensive sampling of soil communities undertaken in this study encompassed gradients at multiple spatial scales allowing us to rigorously test the spatial dependence of environmental sorting and revealed complex patterns which would have been absent or obscured by less extensive sampling and analysis. Performing this work in a simplified and relatively stable setting lacking higher plants and animals also allowed us to assess the environmental controls on microbial communities while limiting complex inter-domain interactions. The results from our analysis highlight the importance and complexity of species sorting processes both within and across scales: microbial biomass, 16 S rRNA gene richness, and bacterial community structure and composition were all differentially related to a variety of environmental characteristics at both local and regional scales with significant evidence of contextual effects at both scales suggesting scale dependent variation in underlying mechanisms. These findings have significant implications for the interpretation of microbial/environmental relationships, as extrapolating patterns or mechanisms between scales is likely to obscure complexities and lead to biased conclusions.

## Supporting Information

Figure S1 **Rarefaction curves for each sample depicting the observed OTUs (97% similarity) for a given sequencing effort.**
(TIF)

Figure S2 **Boxplots of the ln of the water content (mg water g^−1^ dry soil) found in exposed control (C) and snow patch associated (P) soil samples.**
(TIF)

Text S1 **A detailed description of the contextual effects and the mixed effects modeling.**
(DOCX)
