# Supplementary material for: Factors Controlling Soil Microbial Biomass and Bacterial Diversity and Community Composition in a Cold Desert Ecosystem: Role of Geographic Scale
Source: PLoS One. 2013 Jun 18;8(6):e66103. doi: 10.1371/journal.pone.0066103 (PMC3688848; doi:10.1371/journal.pone.0066103)
Supplement: Text S1 — A detailed description of the contextual effects and the mixed effects modeling. (DOCX) [file pone.0066103.s003.docx]

**Supplementary Information:**

**S1:**

Mixed effects models were used which allow inferences to be made beyond the basins sampled in this study. We began by using graphs to examine the relationships between predictors and outcomes both across and within basins. The strength of these relationships (Figure 3) as well as the direction (Figure 2 and 4) sometimes differed between and across basins. These results suggest the use of more complex specifications of the mixed effects model, which enable the examination of effects within and between contexts and allow for effects to differ across contexts.

In the notation of Raudenbush and Bryk [[1](#_ENREF_1)] the model used is:

Level-1 (between samples within basins):

$Y_{ij}=\beta_{0j}+\beta_{1j}X_{ij}+r_{ij}$ Eq. 1

$r_{ij}\sim N(0,\sigma^{2})$ Eq. 2

Level-2 (between basins):

$\beta_{0j}=\gamma_{00}+\gamma_{01}\left( \bar{X}_{Basinj} \right)+u_{0j}$ Eq. 3

$\beta_{1j}=\gamma_{10}+u_{1j}$ Eq. 4

$u_{0j,}u_{1j}\sim N(0,\Sigma)$ Eq. 5

Where Y*_ij_* is the observed value of the outcome for sample *i* in basin *j*, and X*_ij_* is the predictor. β_1_*_j_* is the effect of X for basin *j*, and β_0_*_j_* is intercept for basin *j*, which can be interpreted as the expected value of the outcome when the predictor value (X) is equal to the grand centered mean.

Because these models allow for basin specific values of both the intercept and slope they are ideal for modeling effects such as those seen in Figure 2 and 4 where the relationship of Y to X appears to differ across basins (local contextual effect). These relationships are explicitly modeled at level two. The slope for each basin, β_1_*_j_*, is modeled as a function of the fixed ‘average’ effect across all basins, γ_10_, and a random effect, *u*_1_*_j_*, which allows each basin to differ from this average effect. For the intercepts we also estimate the fixed effect γ_00_ and a random effect *u*_0_*_j_*. However, the effect of the basin mean (γ_01_) is now incorporated into the model, which asks whether there are differences in intercepts between basins as a function of the mean level of the predictor in each basin. Because the intercept is adjusted for levels of the predictor in equation 1, the question answered becomes ‘do basin means have an impact on outcomes beyond the impact observed within each basin’. This is interpreted as a regional effect asking whether basin levels of the predictor impact the outcome beyond what would be expected given the average effect of the predictor across basins [[1](#_ENREF_1)]

A very important aspect of this model is that the random errors are decomposed into their component parts. The within basin error is *r_ij_* which is assumed to be normal and independent and has a variance σ^2^. The between basin error terms represent the difference of each basin’s slope from the average slope across basins (*u*_0_*_j_*) and the difference of the intercept in each basin from the average (*u*_1_*_j_*) conditional on the observed mean level of the predictor within that basin. These errors are also assumed to be independent and normally distributed with a mean zero and a variance/covariance matrix Σ (Eq. 5). The variance of the intercept Σ_00_ shows how much variability there is between basins from the average intercept. This term is always quite large in these models indicating that the conditional mean of the outcome varies substantially across basins. Inclusion of this term is necessary to obtain accurate standard errors and so this term is included in all models. A variance of the slope Σ_11_ that is not different from zero indicates that the effects of the level 1 predictor β*_1j_* do not vary across basins. Additionally, the covariance of the intercept and slope Σ_01_ indicates that there is a correlation between intercepts and slopes across basins (for example a positive correlation indicates that basins with higher conditional means also have higher slopes).

It is important that the random effects portion of the model is correctly specified before interpreting the ‘average’ fixed effects because if effects differ between basins the average effect across basins has a different interpretation than if the effects are homogeneous. Each analyses began by specifying the random effects portion of the model. Using Restricted Maximum Likelihood Estimation (REML) we tested if the variance of the slope (Σ_11_) differed between basins indicating the presence of significant differences between basins in slopes. If this was found we also tested whether the covariance of the intercept and slope (Σ_01_) was different from zero providing evidence that intercepts and slopes are correlated. Because of the limited number of basins available for these analyses we use an alpha level of 0.10 which in this case provides a reasonable tradeoff between power and type I errors.

Once the correct fixed effects were specified we then tested the ‘average’ random effects. Tests of statistical significance were obtained by comparing the log likelihood (LL) value of models without each fixed effect to the LL of the model with that effect, the result is chi-square distributed with 1 df and tests the null hypothesis that the average fixed effect is equal to zero [[2](#_ENREF_2)].

**References**

1. Raudenbush SW, Bryk AS (2002) Hierarchical linear models: applications and data analysis methods. Thousand Oaks: Sage Publications. 512 p.

2. Pinheiro J, Bates D (2009) Mixed-effects models in S and S-PLUS. New York: Springer Verlag. 548 p.
